# Karnofsky Performance Status (KPS) ≤60 Is Strongly Associated With Shorter Brain-Specific Progression-Free Survival Among Patients With Metastatic Breast Cancer With Brain Metastases

**DOI:** 10.3389/fonc.2022.867462

**Published:** 2022-07-27

**Authors:** Mark Freeman, Marguerite Ennis, Katarzyna J. Jerzak

**Affiliations:** ^1^ Faculty of Medicine, University of Toronto, Toronto, ON, Canada; ^2^ Independent Researcher, Markham ON, Canada; ^3^ Sunnybrook Health Science Centre, University of Toronto, Toronto, ON, Canada; ^4^ Sunnybrook Research Institute (SRI), Toronto, ON, Canada

**Keywords:** breast cancer, brain-specific progression-free survival (bsPFS), progression free survival (PFS), overall survival (OS), leptomeningeal disease, karnofsky performance scale (KPS), prognostic marker

## Abstract

**Objective:**

To examine the association between Karnofsky Performance Status (“KPS”) and brain-specific progression-free survival (“bsPFS”) among patients with breast cancer brain metastases (“BCBrM”).

**Methods:**

Using a previously compiled retrospective cohort of 683 patients who were treated for BCBrM with surgery and/or radiotherapy at the Sunnybrook Odette Cancer Centre from 2008-2018, electronic records were reviewed to impute KPS scores at the time of BCBrM diagnosis. Patients were then grouped into KPS ≤60 and KPS >60 cohorts. The dataset was analyzed to identify variables that were prognostic for bsPFS and/or overall survival (“OS”) using univariable and multivariable Cox proportional hazards models.

**Results:**

The mean age of patients was 57 (range 24-93). Most patients (*n*=622, 91%) had extracranial metastatic disease and 174 (25%) had leptomeningeal disease. 247 patients (36%) had hormone receptor (“HR”)-positive/human endothelial growth factor receptor 2 (“HER2”)-negative tumours, 189 (28%) had HER2-positive disease, and 153 (22%) had triple-negative breast cancer. Of the 331 patients (48%) who could be assigned a KPS cohort, 102 (31%) had KPS ≤60. Most patients were treated with whole brain radiotherapy (*n*=498, 73%) and/or stereotactic radiosurgery (“SRS”) (*n*=128, 19%). Median bsPFS was 9 months (95% CI 8-10 months) and median OS was not reached. In univariable analyses, KPS ≤60, presence of leptomeningeal disease, neurological symptoms, ≥2 brain metastases, and not undergoing SRS were factors associated with shorter bsPFS. In a multivariable analysis, KPS ≤60 was the only statistically significant determinant of bsPFS (HR 1.86, 95% CI 1.20-2.88). Although survival data was limited, KPS ≤60 was associated with shorter OS in both univariable (HR 3.12, 95% CI 1.85-5.26) and multivariable (HR 2.95, 95% CI 1.55-5.58) analyses.

**Conclusion:**

Patients with BCBrM who have a KPS ≤60 have significantly shorter bsPFS and OS than those with KPS >60. KPS should be documented routinely at the time of diagnosis of brain metastases to improve prognostication.

## Introduction

In the past two decades, there have been major advances in systemic therapies for patients with metastatic breast cancer (“MBC”), particularly among those with human endothelial growth factor receptor 2 (“HER2”) positive disease (1). In addition, the advent of stereotactic radiosurgery (“SRS”) has revolutionized locoregional treatment of breast metastases (2, 3). The end result of these and other developments has been improved survival for patients with metastatic breast cancer (2–4).

Unfortunately, patients with MBC and brain metastases (“BrM”) have poor outcomes compared to those without BrM (5–9). While blood-brain-barrier-crossing HER2-targeted therapies (10) and SRS (2, 3, 11) have improved outcomes for these patients, they continue to suffer from high rates of morbidity and mortality (12, 13), often driven by neurocognitive sequelae of their intracranial metastatic disease (14–16).

Patients with BrM had historically been excluded from many clinical trials in oncology, and this patient population remains understudied despite improving outcomes with advances in cancer treatment (8, 17). Accordingly, there is growing interest in understanding clinical outcomes of patients with breast cancer BrM above and beyond quantification of overall survival, which is the outcome of interest in most prognostic tools, including the Updated Breast Graded Prognostic Assessment (“BreastGPA”) (8). In a multinational cohort of 2473 patients enrolled from 2006-2017, factors associated with worse overall survival from the time of diagnosis of breast cancer brain metastases (“BCBrM”) included KPS (18, 19) (≤60 vs 70-80 vs 90-100), age (≥60 vs <60), extracranial metastases (presence vs absence), number of BrM (≥2 vs 1), and molecular subtype (HER2 vs luminal B vs luminal A vs basal) (8). However, brain-specific progression-free survival ("bsPFS") was not assessed and patients with leptomeningeal disease were not included in that study.

The relative lack of understanding of bsPFS prognostication represents a clinically important knowledge-gap in the management of patients with BCBrM, particularly because intracranial progression may cause neurological symptoms and/or loss of cognitive function. Further, neurologic progression of disease is not necessarily a cause of death among patients with metastatic breast cancer; in fact, only ~50% of patients with HER2+ BCBrM die of intracranial disease (14). Here we evaluated the prognostic associations of KPS with both bsPFS and OS in a large, single-centre cohort of patients with BCBrM and/or leptomeningeal metastases.

## Methods

### Study Design and Population

We used a previously established cohort of 683 individuals who underwent radiotherapy and/or metastasectomy for BrM from 2008-2018 at Sunnybrook Health Sciences Centre in Toronto, Canada. Details regarding the cohort and baseline characteristics of included patients have been previously outlined (9). All factors found to be significantly associated with bsPFS or OS in the Sperduto et al. (8) or Gao et al. ( 9) studies were included in this analysis. These included KPS, age, tumour receptor status, presence of leptomeningeal disease, presence of neurological symptoms, number of BrM, location of extracranial metastases, and initial treatment modality for BrM. As KPS was not originally studied in our cohort, methods for imputing KPS are outlined in **Supplementary Table 1**.

Given the importance of the BreastGPA in clinical prognostication of OS in BCBrM, we sought to include all prognostic variables that are included in the BreastGPA and treat them as similarly as possible to the way they were treated in Sperduto et al. ( 8 *)* That paper cohorted KPS into cohorts of 90-100, 70-80, and ≤60. Our dataset was felt not to be large enough and available estimates of KPS not precise enough to be able to do useful analysis on a three-cohort KPS division as done in Sperduto et al. Accordingly, we mimicked their KPS ≤60 cohort and combined their KPS 90-100 and KPS 70-80 cohorts into a single KPS >60 cohort for our analysis.

### Statistical Analysis

For the purposes of our study, we defined bsPFS as the length of time from the date of diagnosis of brain metastases to the date of either disease progression in the brain (as determined based on a combination of radiologic and clinical assessment) or death due to any cause, whichever was sooner. Overall survival (OS) was defined as the length of time from the date of diagnosis of brain metastases to the date of death due to any cause.

The Kaplan-Meier method (20) was used to estimate both bsPFS and OS, with comparison of the low (≤60) versus high (>60) KPS groups using the log-rank test. Univariable and multivariable analyses of bsPFS and OS were performed with Cox proportional hazards models (21). All variables which were prognostic for bsPFS and/or OS in a univariable model (*p*-value <0.05) were aggregated into a multivariable Cox proportional hazards model (21) for that outcome. Patients for whom data was missing for any of the statistically significant variables from the univariable analysis were omitted from the multivariable analysis. Variables with *p*-values <0.05 in the multivariable model of an outcome were considered significant predictors of that outcome. All statistics were computed using RStudio Version 1.4.1106.

This study received approval from Sunnybrook Research Institute’s Research Ethics Board.

## Results

### Population Characteristics

Data regarding 13 explanatory variables of interest were summarized (**Table 1**). Information about patient age and initial locoregional treatment modality for BrM was available for all patients. Values for most other explanatory variables were available for 95% or more of the patients in the dataset; the exceptions were tumor receptor status, presence of multiple BrM, and KPS score, with 14%, 28%, and 52% missing data respectively. The great majority of patients (91%) had extracranial metastatic disease, with bone metastases being the most common individual site (68%). 25% of patients had leptomeningeal disease. Patients both above and below age 60 were well-represented (40% and 60% respectively). In our cohort, 36% (*n*=247) of patients had HR+HER2- breast cancer, 28% (*n*=189) had HER2+ and 22% (*n*=153) had triple-negative breast cancer. Most patients (73%) underwent whole-brain radiotherapy (“WBRT”), and a minority received SRS or required neurosurgery (19% and 10% respectively). Notably, 38 patients (6%) received more than one initial therapy for BrM. **Table 1**.

**Table 1:** Characteristics of the study population.

	All patients		KPS not cohortable		KPS cohortable		*p*-value	KPS >60		KPS ≤60		*p-value*
***Study Population***												
	683	(100%)	352	(52%)	331	(48%)		229	(69%)	102	(31%)	
***Extracranial Metastasis***												
Yes	622	(91%)	315	(89%)	307	(93%)	0.18	214	(93%)	93	(91%)	0.38
No	49	(7%)	28	(8%)	21	(6%)		14	(6%)	7	(7%)	
Unknown	12	(2%)	9	(3%)	3	(1%)		1	(0%)	2	(2%)	
***Bone Metastasis***												
Yes	462	(68%)	222	(63%)	240	(73%)	0.02	169	(74%)	71	(70%)	0.14
No	197	(29%)	114	(32%)	83	(25%)		57	(25%)	26	(25%)
Unknown	24	(4%)	16	(5%)	8	(2%)		3	(1%)	5	(5%)
***Lung Metastasis***												
Yes	382	(56%)	171	(49%)	211	(64%)	5 × 10^-5^	158	(69%)	53	(52%)	0.01
No	271	(40%)	158	(45%)	113	(34%)		67	(29%)	46	(45%)
Unknown	30	(4%)	23	(7%)	7	(2%)		4	(2%)	3	(3%)
***Liver Metastasis***												
Yes	362	(53%)	170	(48%)	192	(58%)	0.02	132	(58%)	60	(59%)	0.51
No	287	(42%)	159	(45%)	128	(39%)		91	(40%)	37	(36%)
Unknown	34	(5%)	23	(7%)	11	(3%)		6	(3%)	5	(5%)
***Lymph Node Metastasis***												
Yes	419	(61%)	216	(61%)	203	(61%)	0.39	145	(63%)	58	(57%)	0.38
No	234	(34%)	117	(33%)	117	(35%)		78	(34%)	39	(38%)
Unknown	30	(4%)	19	(5%)	11	(3%)		6	(3%)	5	(5%)
***Leptomeningeal Disease***												
Yes	174	(25%)	82	(23%)	92	(28%)	0.16	61	(27%)	31	(30%)	0.10
No	485	(71%)	254	(72%)	231	(70%)		165	(72%)	66	(65%)
Unknown	24	(4%)	16	(5%)	8	(2%)		3	(1%)	5	(5%)
***Multiple Brain Metastases***												
Yes	382	(56%)	192	(55%)	190	(57%)	0.14	131	(57%)	59	(58%)	0.70
No	108	(16%)	65	(18%)	43	(13%)		32	(14%)	11	(11%)
Unknown	193	(28%)	95	(27%)	98	(30%)		66	(29%)	32	(31%)
***Tumour Subtype***												
HR+HER2-	247	(36%)	119	(34%)	128	(39%)	0.02	89	(39%)	39	(38%)	0.01
HER2+	189	(28%)	91	(26%)	98	(30%)		76	(33%)	22	(22%)
TNBC	153	(22%)	80	(23%)	73	(22%)		49	(21%)	24	(24%)
Unknown	94	(14%)	62	(18%)	32	(10%)		15	(7%)	17	(17%)
***Age***												
≥60	275	(40%)	140	(40%)	135	(41%)	0.85	82	(36%)	53	(52%)	0.01
<60	408	(60%)	212	(60%)	196	(59%)		147	(64%)	49	(48%)
***Neurological Symptoms***												
Yes	529	(77%)	270	(77%)	259	(78%)	0.02	165	(72%)	94	(92%)	2 × 10^-4^
No	119	(17%)	55	(16%)	62	(19%)		56	(24%)	6	(6%)
Unknown	37	(5%)	27	(8%)	10	(3%)		8	(3%)	2	(2%)
***Stereotactic Radiosurgery***												
Yes	128	(19%)	52	(15%)	76	(23%)	0.01	70	(31%)	6	(6%)	2 × 10^-6^
No	555	(81%)	300	(85%)	255	(77%)		159	(69%)	96	(94%)
***Neurosurgery***												
Yes	69	(10%)	43	(12%)	26	(8%)	0.08	22	(10%)	4	(4%)	0.12
No	614	(90%)	309	(88%)	305	(92%)		207	(90%)	98	(96%)
***Whole Brain Radiotherapy***												
Yes	498	(73%)	268	(76%)	230	(69%)	0.06	143	(62%)	87	(85%)	5 × 10^-5^
No	165	(27%)	84	(24%)	101	(31%)		86	(38%)	15	(15%)

Entries compare the prevalence and proportions of patient characteristics analyzed in this paper among all study patients, study patients who could not be assigned to a Karnofsky Performance Status (“KPS”) cohort (referred to in the table as “KPS uncohortable” patients), patients who were assigned to a KPS cohort (“KPS cohortable” patients), patients who were assigned to the high-KPS cohort, and patients assigned to the low-KPS cohort, respectively. P-values in the 8^th^ column from the left refer to the statistical significance of χ^2^-tests for differences in proportions between the populations of patients who could and could not be assigned KPS cohorts, and the p-values in the rightmost column refer to the statistical significance of χ ^2^-tests for differences in proportions between the KPS ≤60 and KPS >60 populations. Low KPS is statistically significantly associated with both shorter bsPFS and shorter OS in univariable models.

### Karnofsky Performance Status Subset Analysis

Some patients’ charts did not contain sufficient information to determine whether their KPS was ≤60 or >60 at the time of diagnosis of brain metastases (see ‘Supplementary Methods’ section below). Characteristics were compared between the “KPS cohortable” patients to whom a KPS cohort could be assigned and the “KPS not cohortable” patients to whom a KPS cohort could not be assigned (see **Table 1** above). There were some statistically significant differences between the two groups; the KPS cohortable patients were more likely to have bone (73% vs 63%), liver (58% vs 48%), and lung (64% vs 49%) metastases. They were also more likely to undergo treatment with SRS (23% vs 15%), and to be free of neurological symptoms at the time of diagnosis of BrM (19% vs 16%). Finally, two groups had slightly different distributions of tumour subtypes, with KPS cohortable patients slightly more likely to have HR+HER2- (39% vs 34%) and HER2+ (30% vs 26%) disease compared to their KPS non-cohortable counterparts.

Within the KPS cohortable group, there were important statistically significant differences between the patient populations in the high-KPS and low-KPS cohorts. Patients with high KPS were more likely to be under the age of 60 (64% vs 48%) and more likely to have lung metastases (69% vs 52%); they were also more likely to have asymptomatic BrM (24% vs 6%) and more likely to receive SRS (31% vs 6%) compared to the low-KPS cohort.

### Univariable Analyses for bsPFS and OS

**Figure 1:**
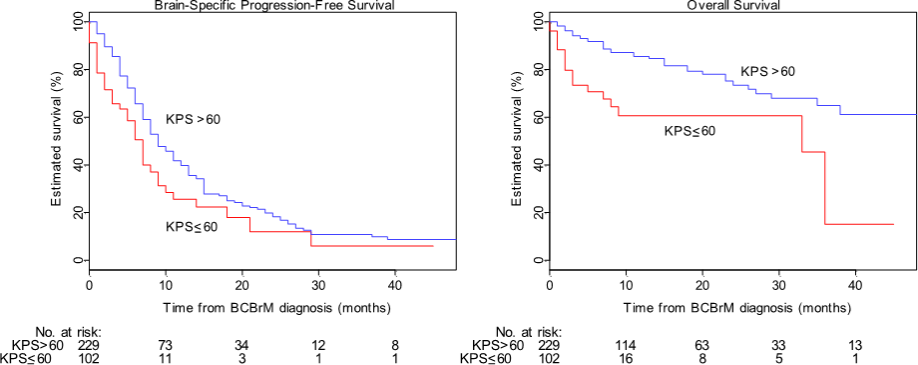
Brain-specific progression-free survival (“bsPFS”) and overall survival (“OS”) by Karnofsky Performance Status (“KPS”) cohort. Survival plots are shown of the low-KPS (red) and high-KPS (blue) cohorts for bsPFS (left) and OS (right). Low KPS is statistically significantly associated with both shorter bsPFS and shorter OS in univariable models.

Overall median bsPFS for the population was 9 months (95% CI: 8-10 months) with median follow-up 13 months (95% CI: 10-15 months). A univariable analysis identified presence of leptomeningeal disease, KPS ≤60, presence of neurological symptoms, and not undergoing SRS as significantly associated with shorter bsFPS (see **Table 2** for details). Of these, KPS ≤60 was most strongly associated with shorter bsPFS with a hazard ratio of 1.64 (95% CI 1.16-2.33). KM curves for bsPFS by KPS are shown in **Figure 1**.

**Table 2:** Cohort sizes and effect on hazard ratio (“HR”) for overall survival (“OS”) and brain-specific progression-free survival (“bsPFS”) for all explanatory variables studied.

	*n*	HR for bsPFS(95% CI)	univariable *p*-value for bsPFS	HR for OS (95% CI)	univariable *p*-value for OS
Extracranial Metastasis (any vs none)	671	0.97 (0.66, 1.45)	0.9	1.13 (0.56, 2.27)	0.7
Bone Metastasis (any vs none)	659	1.00 (0.79, 1.27)	1	1.18 (0.77, 1.82)	0.4
Lung Metastasis (any vs none)	653	1.15 (0.92, 1.43)	0.2	1.71 (1.14, 2.57)	0.008
Liver Metastasis (any vs none)	649	1.10 (0.89, 1.37)	0.4	1.91 (1.28, 2.85)	0.001
Lymph Node Metastasis (any vs none)	653	0.91 (0.73, 1.14)	0.4	1.32 (0.88, 1.99)	0.2
Leptomeningeal Disease (yes vs no)	659	1.34 (1.06, 1.69)	0.01	1.50 (1.01, 2.23)	0.04
Multiple Brain Metastases (yes vs no)	490	1.43 (1.04, 1.96)	0.02	1.56 (0.87, 2.80)	0.1
HER2+ Tumour (yes vs no)	589	0.81 (0.65, 1.02)	0.07	0.51 (0.33, 0.78)	0.001
Triple Negative Tumour (yes vs no)	589	1.28 (0.99, 1.65)	0.05	1.56 (1.01, 2.41)	0.04
Age (≥60 vs <60)	683	1.23 (0.99, 1.53)	0.06	1.71 (1.17, 2.48)	0.004
KPS Score (≤60 vs >60)	331	1.64 (1.16-2.33)	0.003	3.12 (1.85, 5.26)	6 × 10^-6^
Neurological Symptoms (any vs none)	646	1.32 (1.01, 1.74)	0.04	1.76 (1.02, 3.02)	0.03
Stereotactic Radiosurgery (yes vs no)	683	0.77 (0.60, 0.99)	0.03	0.40 (0.24, 0.69)	4 × 10^-4^
Neurosurgery (yes vs no)	683	0.90 (0.66, 1.23)	0.5	0.27 (0.11, 0.66)	0.002
Whole-brain Radiotherapy (yes vs no)	683	1.23 (0.98, 1.54)	0.06	2.77 (1.72, 4.46)	8 × 10^-6^

Cohort sizes listed for each variable exclude patients for whom data for that variable was unavailable.

Median OS in the overall population was not reached; median follow-up for OS was 7 months (95% CI: 6-9 months). Presence of lung metastases, liver metastases, leptomeningeal disease, and neurological symptoms at time of diagnosis of BrM were significantly associated with shorter survival, as were KPS ≤60, age ≥60, and not receiving SRS or neurosurgery (**Table 2**).

**Table 3:** Results of multivariable data analysis.

	HR for bsPFS(95% CI)	multivariable *p*-value for bsPFS	HR for OS (95% CI)	multivariable *p*-value for OS
Lung Metastasis (any vs none)			1.35 (0.72, 2.51)	0.34
Liver Metastasis (any vs none)			1.95 (1.06, 3.60)	0.03
Leptomeningeal Disease (yes vs no)	1.34 (0.91, 1.99)	0.13	1.67 (0.91, 3.07)	0.09
Multiple Brain Metastases (yes vs no)	1.48 (0.92, 2.40)	0.10		
HER2+ Tumour (vs HR+HER2- tumour)			0.62 (0.32, 1.21)	0.15
Triple Negative Tumour (vs HR+HER2- tumour)			1.68(0.85, 3.30)	0.12
Age (≥60 vs <60)			1.41 (0.80, 2.50)	0.22
Karnofsky Performance Status (≤60 vs >60)	1.86 (1.20-2.88)	0.005	2.95 (1.55, 5.58)	7 × 10^-4^
Neurological Symptoms (any vs none)	1.15 (0.74, 1.79)	0.52	1.74 (0.82, 3.73)	0.14
Stereotactic Radiosurgery (yes vs no)	0.94 (0.64, 1.38)	0.76	0.76 (0.22, 2.66)	0.67
Neurosurgery (yes vs no)			0.39 (0.08, 1.86)	0.23
Whole Brain Radiotherapy (yes vs no)			1.09 (0.37, 3.23)	0.88

The multivariable model for each outcome only included variables which were significant predictors of that outcome in a univariable analysis. Blank values in the table indicate variables which were not significant for the outcome in question; variables listed in **Table 2** but not in **Table 3** were not significant in a univariable analysis for either outcome. Each analysis was carried out only on the subset of the patient population which had no missing data for any of the explanatory variables listed in the multivariable model examined. For brain-specific progression-free survival (“bsPFS”), this meant a population of 217 patients with 131 observed events between them; for overall survival (“OS”) this meant a population of 279 with 58 observed events between them.

### Multivariable Analyses

The only variable significant for shorter bsPFS in the multivariable analysis was KPS ≤60 (HR 1.86, 95% CI 1.20-2.88), and the only two variables significant for shorter OS in the multivariable analysis were KPS ≤60 (HR 2.95, 95% CI 1.55-5.58) and the presence of liver metastases (HR 1.95, 95% CI 1.06-3.60).

## Discussion

### Karnofsky Performance Status as a Key Prognostic Indicator Among Patients With Breast Cancer Brain Metastases

Performance status has been repeatedly identified as an important marker of prognosis among patients with breast cancer (8, 22–24). Our study confirms that the prognostic significance of KPS extends to patients with brain metastases from breast cancer, given that a KPS ≤60 was significantly associated with a shorter bsPFS and OS.

Once KPS was accounted for, none of the other variables examined were significantly associated with bsPFS in our multivariable analysis. This is likely due to an intimate connection between KPS and other variables that are associated with bsPFS (9). Increasing age is associated with higher burdens of comorbidity and greater frailty, which tend to lead to diminishing functional abilities and therefore lower KPS. Similarly, the presence of neurological symptoms at diagnosis often heralds a greater need for support in activities of daily living or even confusion, both of which diminish KPS. This hypothesis is supported by the statistically significant differences in prevalence of age ≥60, neurological symptoms, and lung metastases between high- and low-KPS cohorts in our dataset (see **Table 1** above).

### Concordance of Findings With Previous Research

This study confirms the findings of Sperduto et al. that low KPS is a negative prognostic factor in patients with breast cancer and BrM (8) in a dataset which included a significant proportion of patients with no neurological symptoms at the time of diagnosis of BrM (17%) as well as of patients with leptomeningeal disease (25%). Neither of these latter populations are well represented in the patient population examined by Sperduto et al. ( 8).

Like in Sperduto et al, triple-negative breast cancer subtype and advanced age were associated with shorter OS and HER2-positive breast cancer was associated with longer OS, albeit only in univariable analyses. Given that these variables were previously shown in the larger Sperduto et al. dataset to be associated with OS in a multivariable analysis that included KPS (8), we suspect that our study was underpowered to confirm this.

One noteworthy difference between the results of our study and that of Sperduto et al. is the impact of extracranial metastasis on OS. In the latter study, the presence of any extracranial metastasis was a negative prognostic marker, albeit a less important one than tumour receptor status or KPS. The presence of any extra-cranial metastatic disease was not significantly associated with OS in our study, possibly because the small proportion (7%, *n*=49) of patients who presented with brain-only metastatic disease limited our statistical power to detect such an association. In contrast to the BreastGPA, we were able to refine our analysis by metastatic site. The presence of lung and/or liver metastases were individually prognostic for shorter OS in univariable analyses, and the presence of liver metastases was significantly associated with shorter OS in a multivariable model. The impact of location and burden of extracranial metastatic disease on clinical outcomes requires further study in larger datasets.

### Study Limitations

Unfortunately, there was a relative scarcity of data suitable for KPS imputation in our study. More than half of the patients did not have enough information available in their medical records to confidently assign them to the low- or high-KPS cohort. The omission of these patients created an “excluded middle” of patients whose absence created a dataset weighted towards high and low KPS, emphasizing the effects of KPS on the outcomes studied. Nonetheless, the distribution of population characteristics between the study population as a whole and those for whom a KPS cohort was available was similar (**Table 1**). Hence, our inability to cohort all patients by KPS is unlikely to have meaningfully altered our results.

Another important caveat is that only a minority of the patients who were successfully cohorted by KPS had explicitly documented performance statuses on any clinically validated scale around the time of diagnosis of BrM. For the remainder, a KPS was calculated indirectly from a PPS. This imputed PPS was often based on only one or two of the criteria evaluated in the PPS metric, with information on the remaining criteria being unavailable. It is likely that some of the patients’ imputed PPS scores would have been different had more complete information been available in the records.

It is also noteworthy that KPS estimation could not be done in a blinded fashion. Bias introduced by the unblinded nature of assigning KPS was mitigated by the adoption of strict criteria delineated in the ‘Supplementary Methods’ section. Finally, the timing of KPS estimation was not perfectly uniform as outlined in **Supplementary Table 1**. Other caveats discussed in Gao et al. regarding the single-academic-centre, retrospective nature of this study and the challenges of applying such a study to other centres with different practice patterns and demographics (9) remain relevant in our study as well.

## Conclusion

Our study confirms that a Karnofsky Performance Status ≤60 is strongly associated with shorter brain-specific progression-free survival as well as shorter overall survival in a large “real world” cohort of patients treated for breast cancer with brain metastases at our institution, with significant representation of patients with leptomeningeal disease [a subgroup which is poorly represented in the population which was used to establish the BreastGPA index (8)]. Despite its clear prognostic importance in this clinical setting, performance status was usually not explicitly documented in patients’ medical records. Performance status of patients who are newly diagnosed with BCBrM should be assessed to improve clinical prognostication and to guide patient discussions in this clinical setting.

## Data Availability Statement

The data analyzed in this study is not freely available due to the following restrictions: patient data confidentiality. Requests to access these datasets should be directed to the corresponding author.

## Ethics Statement

The studies involving human participants were reviewed and approved by Sunnybrook Research Institute Ethics Board, Sunnybrook Research Institute, Toronto, ON, Canada. Written informed consent for participation was not required for this study in accordance with the national legislation and the institutional requirements.

## Author Contributions

MF collected the KPS data, wrote scripts to run the data analysis, and wrote most of the manuscript. ME helped design and critique the data analysis in the project and proofread the manuscript. KJ supplied the other data used in the paper, designed the study, supervised research efforts, and proofread the manuscript. All authors contributed to the article and approved the submitted version.

## Conflict of Interest

Author KJJ reports the following: Speaker/advisor board/consultant for: Amgen, Apo Biologix, AstraZeneca, Eli Lilly, Esai, Exact Sciences, Knight Therapeutics, Pfizer, Seagen, Merck, Novartis, Roche. Research funding: Eli Lilly, Astra Zeneca, Seagen. Author ME reports personal fees from Mount Sinai Hospital, London Health Sciences Centre and Sunnybrook Health Sciences Centre for statistical consulting.

The remaining authors declare that the research was conducted in the absence of any commercial or financial relationships that could be construed as a potential conflict of interest.

## Publisher’s Note

All claims expressed in this article are solely those of the authors and do not necessarily represent those of their affiliated organizations, or those of the publisher, the editors and the reviewers. Any product that may be evaluated in this article, or claim that may be made by its manufacturer, is not guaranteed or endorsed by the publisher.
