# Supplementary material for: Karnofsky Performance Status (KPS) ≤60 Is Strongly Associated With Shorter Brain-Specific Progression-Free Survival Among Patients With Metastatic Breast Cancer With Brain Metastases
Source: Front Oncol. 2022 Jul 27;12:867462. doi: 10.3389/fonc.2022.867462 (PMC9364681; doi:10.3389/fonc.2022.867462)
Supplement: Supplementary file 1 [file DataSheet_1.pdf]

SUPPLEMENTARY METHODS

Rules for Imputation of Karnofsky Performance Status

For each patient, a KPS score at the time of diagnosis of brain metastases was imputed from their medical records, using the following rules:

1. Information from the patient's medical record was considered for the purposes of KPS imputation if it was published within the "KPS window". This is a timeframe defined as starting 3 months prior to the date of diagnosis of the brain metastases. The window was defined to end when either 1 month had elapsed from the date of diagnosis or the patient had already received radiotherapy or surgery to treat the brain metastases, whichever was sooner. Of note, since a large fraction of patients received dexamethasone within hours of diagnosis of their brain metastases, a record published after a patient received medical therapy for treatment of their brain metastases was still considered usable for the purposes of KPS estimation.
2. If a patient was explicitly assigned a KPS score in a medical document published within the KPS window, that score was used as the patient's KPS score for the purposes of our study.
3. If a patient's performance status had previously been quantified with either the Palliative Performance Scale ("PPS")[25] or Eastern Cooperative Oncology Group ("ECOG")[26, 27] score in a medical document published within the KPS window, and that score was not obviously contradicted by information elsewhere in that same document, that score was converted to a KPS score in accordance with a previously validated conversion table[28] (see Supplementary Table 1 below).
4. If no medical document published within the KPS window contained an explicit and credible performance status score, other information in the medical record from documents published within the KPS window was used to estimate a range of possible PPS scores in accordance with Supplementary Table 1 below. This estimated PPS score was then converted by the same table to the corresponding KPS score.
5. If there was no evidence in the patient's chart within the KPS window which could be used to assign a narrow range of KPS values to the patient, no KPS score was assigned to the patient.
