# Supplementary material for: Karnofsky Performance Status (KPS) ≤60 Is Strongly Associated With Shorter Brain-Specific Progression-Free Survival Among Patients With Metastatic Breast Cancer With Brain Metastases
Source: Front Oncol. 2022 Jul 27;12:867462. doi: 10.3389/fonc.2022.867462 (PMC9364681; doi:10.3389/fonc.2022.867462)
Supplement: Supplementary file 2 [file Table_1.pdf]

<table>
<tr><th>KPS</th><th>PPS</th><th>ECOG</th><th>Ambulation</th><th>Activity</th><th>Self-care</th><th>PO intake</th><th>Cognition</th></tr>
<tr><td>100</td><td>100</td><td>0</td><td rowspan="3">full</td><td>normal; no evidence of disease</td><td rowspan="4">full</td><td rowspan="2">full</td><td rowspan="4">full</td></tr>
<tr><td>90</td><td>90</td><td rowspan="2">1</td><td>normal; some evidence of disease</td></tr>
<tr><td>80</td><td>80</td><td>normal with effort; some evidence of disease</td><td rowspan="6">full or reduced</td></tr>
<tr><td>70</td><td>70</td><td rowspan="2">2</td><td rowspan="2">reduced</td><td>unable to do job</td></tr>
<tr><td>60</td><td>60</td><td>unable to do hobby/housework</td><td>occasional assistance</td><td rowspan="2">full or confused</td></tr>
<tr><td>50</td><td>50</td><td rowspan="2">3</td><td>mainly sitting</td><td>unable to do any work</td><td>considerable assistance</td></tr>
<tr><td>40</td><td>40</td><td>mainly lying down</td><td rowspan="4">unable to do most activity</td><td>mainly assistance</td><td rowspan="3">full, confused, or drowsy</td></tr>
<tr><td>30</td><td>30</td><td rowspan="3">4</td><td rowspan="3">bedbound</td><td rowspan="3">total care</td></tr>
<tr><td>20</td><td>20</td><td>minimal</td></tr>
<tr><td>10</td><td>10</td><td>mouth care only</td><td>confused, drowsy, or comatose</td></tr>
<tr><td>0</td><td>0</td><td>5</td><td colspan="5">dead</td></tr>
</table>

Supplementary Table 1: Calculation of Karnofksy Performance Status (“KPS”) scores. Pieces of information in the same row(s) are considered to represent equivalent KPS scores. Note that Palliative Performance Scale (“PPS”) scores correspond directly to equivalent KPS scores. Equivalent Eastern Cooperative Oncology Group (“ECOG”) are also displayed. The rightmost 5 columns of the table are the 5 components of the PPS score, which was calculated and translated into the corresponding KPS score.
